# Identification of Appropriate Reference Genes for Normalization of miRNA Expression in Grafted Watermelon Plants under Different Nutrient Stresses

**DOI:** 10.1371/journal.pone.0164725

**Published:** 2016-10-17

**Authors:** Weifang Wu, Qin Deng, Pibiao Shi, Jinghua Yang, Zhongyuan Hu, Mingfang Zhang

**Affiliations:** 1 Laboratory of Germplasm Innovation and Molecular Breeding, Institute of Vegetable Science, Zhejiang University, Hangzhou, Zhejiang, P. R. China; 2 Key laboratory of Horticultural Plant Growth, Development & Quality Improvement, Ministry of Agriculture, Hangzhou, Zhejiang, P. R. China; 3 Zhejiang Provincial Key Laboratory of Horticultural Plant Integrative Biology, Hangzhou, Zejiang, P. R. China; Agriculture and Agri-Food Canada, CANADA

## Abstract

Watermelon (*Citrullus lanatus*) is a globally important crop belonging to the family Cucurbitaceae. The grafting technique is commonly used to improve its tolerance to stress, as well as to enhance its nutrient uptake and utilization. It is believed that miRNA is most likely involved in its nutrient-starvation response as a graft-transportable signal. The quantitative real-time reverse transcriptase polymerase chain reaction is the preferred method for miRNA functional analysis, in which reliable reference genes for normalization are crucial to ensure the accuracy. The purpose of this study was to select appropriate reference genes in scion (watermelon) and rootstocks (squash and bottle gourd) of grafted watermelon plants under normal growth conditions and nutrient stresses (nitrogen and phosphorus starvation). Under nutrient starvation, geNorm identified *miR167c* and *miR167f* as two most stable genes in both watermelon leaves and squash roots. *miR166b* was recommended by both geNorm and NormFinder as the best reference in bottle gourd roots under nutrient limitation. Expression of a new *Cucurbitaceae* miRNA, *miR85*, was used to validate the reliability of candidate reference genes under nutrient starvation. Moreover, by comparing several target genes expression in qRT-PCR analysis with those in RNA-seq data, *miR166b* and *miR167c* were proved to be the most suitable reference genes to normalize miRNA expression under normal growth condition in scion and rootstock tissues, respectively. This study represents the first comprehensive survey of the stability of miRNA reference genes in *Cucurbitaceae* and provides valuable information for investigating more accurate miRNA expression involving grafted watermelon plants.

## Introduction

Nitrogen (N) and phosphorus (P) are the two most important macronutrients for the growth and development of plants. N is an important constituent of genetic components such as nucleic acids, amino acids, various metabolic compounds including ATP, as well as electron-transfer molecules such as NAD(P) or FAD. It is also involved in the composition of critical elements for photosynthesis: proteins, chlorophyll and pigment. The other crucial component of plant cell structure is phosphorus, participating in energy metabolism and signal transduction cascades and regulating the enzymes activities. For example, the reversible processes of protein phosphorylation and de-phosphorylation play a central role in the transduction of cellular signals and regulation of protein activity [1]. Plants need sufficient N and P uptake from the soil in order to grow and develop vigorously. However, the availability of the nutrients in the soil solution does not always meet the requirement of plants, leading to a detrimental effect on plant growth and development [2–5].

Grafting, an ancient technique, is the unification of different parts of two or more living plants that ultimately grow together as a single plant [6]. Over the past few years, grafting has been widely adopted as an effective and common method for improving the ability of a plant to resist various biotic and abiotic stresses, as well as for enhancing the nutrient uptake efficiency [7]. Watermelon (*Citrullus lanataus*), a crop of high economic value, is usually grafted onto squash (*Cucurbita moschata*) and bottle gourd (*Lagenaria siceraria*). This practice was initially adopted in Japan in the late 1920s and helped to control declining yields due to soil-borne diseases [8,9]. China produces more than half of the world’s watermelons (http://faostat.fao.org), and approximately 20% of these are grafted [6,7].

Recently, the several endogenous miRNAs—20–24 nt single-stranded RNA molecules that can regulate gene expression transcriptionally and post-transcriptionally—have been attributed as regulatory factors during plant responses to N and P deficiency by their regulation of various effector genes [10–14]. Additionally, some miRNAs, such as *miR399*, were found to be graft-transmissible and were seen to regulate N and P homeostasis by acting as phloem-mobile signals [15]. This involvement of miRNAs in grafted watermelon has not been well studied and understanding their function in grafted watermelon in response to nutrient stresses is of great importance. Investigation of expression patterns has provided a crucial means to expand our understanding of the miRNA-mediated molecular regulation during N and P stresses. Quantitative real-time reverse transcriptase polymerase chain reaction (qRT-PCR) is currently one of the most rapid and sensitive techniques for analyzing gene expression profiles [16]. Accurate results can be obtained with this approach depending on the rigid transcript normalization strategy using appropriate reference genes. However, on the contrary, the use of inappropriate reference genes would cause drastic misinterpretation of the gene expression pattern. To avoid the effects of non-biological variation on the results, the suitable reference genes should be assayed together with the genes of interest during qRT-PCR assays. Therefore, the selection of appropriate reference genes to normalize gene expression becomes an important challenge in this technology. At present, a number of appropriate reference genes for miRNAs qRT-PCR analyses are known in plants, such as soybean [17], wheat [18], longan tree [19], watermelon [20] and rapeseed [21]. Wang et al. [22] demonstrated that *PP2A-2* and *UBC* were the most suitable internal reference genes for miRNA expression normalization during bud developmental and the flowering process in different *Prunus mume* genotypes. However, there are few reference genes that can be constitutively expressed under varying growth conditions in different plants.

The expression stability of seventeen candidate reference genes were tested in this study to identify proper reference genes for reviewing the expression of miRNAs in the scion and rootstock of grafted watermelon under different nutrient conditions. The candidate gene expression stability and the most suitable reference gene were determined by further calculations with geNorm and NormFinder. In addition, the expression of several target miRNAs was investigated to validate the efficacy of the selected reference genes. Based on the results obtained, three miRNA reference genes (*miR167c*, *miR167f*, and *miR166b*) were recommended as appropriate for normalization of miRNA expression in grafted watermelon under different nutrition stresses.

## Materials and Methods

### Plant materials, growth conditions, and treatments

Watermelon cultivar 'Zaojia' and two rootstocks: bottle gourd ‘Yongzhen’ and squash ‘Feichangfuzuo’ were the main plant materials used in this research. After germination, all seedlings were grown in the Professional Growing Mix (Fafard^®^ 51L Mix). Grafting was performed when the scion and rootstock were at cotyledon stage and one-leaf-stage, respectively. Self-, squash-, and bottle gourds-grafted watermelons were abbreviated as Wm/Wm, Wm/Sq and Wm/Bg, respectively. Two non-grafted (squash and bottle gourds) plants were also used. All plant materials were grown as described by Liu et al. [9], until the graft at third-true-leaf stage.

For N and P stress treatment, plants were transferred to a hydroponics system containing full-strength Hoagland solution (7.5 mM N and 0.3 mM P, pH = 6) [23] and acclimated for 5 days. The plants were then treated separately with N (0.75 mM) and P (0.01 mM) starvation (S1 Table) for 5 days. The hydroponics solutions were renewed every 3 days to monitor the nutrient concentration. The leaves of different grafted watermelons were collected at the third-leaf-stage, while the root samples were harvested at the same age from the grafted and non-grafted squash and bottle gourd rootstocks. Samples harvested from scion or rootstock after different treatments, were fixed immediately in liquid nitrogen and stored at -80°C until further use.

### Candidate reference gene selection and primer design

As previously mentioned in the present study, seventeen candidate reference genes were selected to identify the most suitable normalizer gene. Among them, eight miRNAs (*miR81*, *miR82*, *miR166b*, *miR170*, *miR3511-3p*, *miR319b*, *miR398b* and *miR166u*) were selected from a high-throughput small RNA sequencing experiment (small RNA-seq) of grafted watermelon plants, as they showed stable expression in respective tissues before and after grafting (S2 Table). Moreover, *miR169* stably expressed in wheat (*Triticum aestivum* L.) and lettuce (*Lactuca sativa* l.) under abiotic stress [18, 24]. *miR167-1_2* was ranked as the best stable reference gene for expression studies during rapeseed (*Brassica napus*) seed development [21]. *miR160a* was selected as a non-nitrogen-regulated miRNA control for *Arabidopsis* (*Arabidopsis thaliana*), showing no nitrogen response [25]. After Blastning in the miRBase 21.0 (http://www.mirbase.org) using these miRNAs mentioned above as queries, five orthologous Cucurbitaceae miRNAs (*miR169n-5p*, *miR167f*, *miR167c*, *miR167b* and *miR160a*) were identified as candidates. Two protein-coding genes *YLS8* and *PP2A* were also reported by Kong et al. [20] and Orebro et al. [26]. In addition, two non-coding RNAs (U6 and 18S) were chosen owing to their historical use in qRT-PCR [27, 28].

The miRNA forward primers were designed based on the mature miRNA sequence and the universal reverse primer was provided by the NCode^™^ VILO^™^ miRNA cDNA Synthesis Kit (Invitrogen). For more comparable results, the primer pairs of protein-coding genes and non-coding RNAs previously published [20, 26, 29] were used in this study. The specificity of the amplification product for each primer pair was determined by the melting curve analysis (S1 Fig). All primer sequences and relevant information pertaining to the reference genes are presented in Table 1.

**Table 1:** Sequence information of the templates and primers for qRT-PCR.

**miRNA**	**Gene description**	**miRNA mature sequence (5'-3')**	**Forward primer (5'-3')**	**Amplication efficiency (%)**		**R**^**2**^	**Reference**
*Cla-miR81*	microRNA	ATGTCTATCTGGGTCTATCGCAGT	ATGTCTATCTGGGTCTATCGCAG	97.916		0.985	Selected from small RNA-seq
*Cla-miR82*	microRNA	ATTGTTGTTACATAAAGGACGAGT	ATTGTTGTTACATAAAGGACGAG	83.609		0.985	Selected from small RNA-seq
*Cla-miR167f*	microRNA	TGAAGCTGCCAGCATGATCTG	TGAAGCTGCCAGCATGATCTG	108.05		0.996	[18,21]
*Cmo-miR167f*	microRNA	TGAAGCTGCCAGCATGATCTG	TGAAGCTGCCAGCATGATCTG	108.127		0.998	[18,21]
*Lsi-miR167f*	microRNA	TGAAGCTGCCAGCATGATCTG	TGAAGCTGCCAGCATGATCTG	109.097		0.998	[18,21]
*Cla-miR166b*	microRNA	TCGGACCAGGCTTCATTCCCC	TCGGACCAGGCTTCATTCCCC	104.928		1	Selected from small RNA-seq
*Cmo-miR166b*	microRNA	TCGGACCAGGCTTCATTCCCC	TCGGACCAGGCTTCATTCCCC	111.222		1	Selected from small RNA-seq
*Lsi-miR166b*	microRNA	TCGGACCAGGCTTCATTCCCC	TCGGACCAGGCTTCATTCCCC	106.805		0.998	Selected from small RNA-seq
*Cla-miR169n-5p*	microRNA	TAGCCAAAAATGACTTGCCTGC	GGTAGCCAAAAATGACTTGCCTGC	102.642		0.993	[24]
*Cla-miR170*	microRNA	TGATTGAGCCGCGCCAATATC	CTGATTGAGCCGCGCCAATATC	104.323		0.998	Selected from small RNA-seq
*Cla-miR167c*	microRNA	TGAAGCTGCCAGCATGATCTT	TGAAGCTGCCAGCATGATCTT	105.961		0.996	[18,21]
*Cmo-miR167c*	microRNA	TGAAGCTGCCAGCATGATCTT	TGAAGCTGCCAGCATGATCTT	103.039		0.998	[18,21]
*Lsi-miR167c*	microRNA	TGAAGCTGCCAGCATGATCTT	TGAAGCTGCCAGCATGATCTT	101.089		0.997	[18,21]
*Cmo-miR3511-3p*	microRNA	AGTTACTAATTAATGATCTGGC	AGTTACTAATTAATGATCTGGC	96.331		0.988	Selected from small RNA-seq
*Cmo-miR167b*	microRNA	TGAAGCTGCCAGCATGATCTA	TGAAGCTGCCAGCATGATCT	107.682		0.997	[18,21]
*Cmo-miR160a*	microRNA	TGCCTGGCTCCCTGTATGCC	TGCCTGGCTCCCTGTATGCC	109.31		1	[25]
*Cmo-miR319b*	microRNA	TGCCTGGCTCCCTGTATGCC	TCGTTGGACTGAAGGGAGC	108.429		0.996	Selected from small RNA-seq
*Lsi-miR398b*	microRNA	TGTGTTCTCAGGTCGCCCCTA	TGTGTTCTCAGGTCGCCCCT	107.615		0.998	Selected from small RNA-seq
*Lsi-miR166u*	microRNA	TCTCGGACCAGGCTTCATTCT	CTCGGACCAGGCTTCATTCTA	109.964		1	Selected from small RNA-seq
*Cmo-miR397a*	microRNA	TCATTGAGTGCAGCGTTGATG	TCATTGAGTGCAGCGTTGATG	114.628		0.991	Selected from small RNA-seq
*Cla-miR164a*	microRNA	TGGAGAAGCAGGGCACGT	TGGAGAAGCAGGGCACGT	93.059		1	Selected from small RNA-seq
*Lsi-miR5148a*	microRNA	GGAGGGGTGCTTGCCTAAGGTCTG	GGAGGGGTGCTTGCCTAAGGTCTG	98.74		0.998	Selected from small RNA-seq
*miR85*	microRNA	AGGACTTTGAAAAGAAAGA	GGAGGACTTTGAAAAGAAAG	105.477		0.987	Selected from small RNA-seq
**universal reverse primer**		**provided with the kit**					
**ncRNA**	**Gene description**	**Forward primer (5'-3')**	**Reverse primer (5'-3')**		**Amplication efficiency (%)**	**R**^**2**^	**Reference**
*Cla-U6*	small nuclear RNA	GGGGACATCCGATAAAATT	TGTGCGTGTCATCCTTGC		101.47	0.998	[28]
*Cmo-U6*	small nuclear RNA	GGGGACATCCGATAAAATT	TGTGCGTGTCATCCTTGC		109.956	0.998	[28]
*Lsi-U6*	small nuclear RNA	GGGGACATCCGATAAAATT	TGTGCGTGTCATCCTTGC		109.798	0.999	[28]
*Cla-18S*	ribosome RNA	AGCCTGAGAAACGGCTACCACATC	ACCAGACTCGAAGAGCCCGGTAT		96.564	0.983	[27]
*Cmo-18S*	ribosome RNA	AGCCTGAGAAACGGCTACCACATC	ACCAGACTCGAAGAGCCCGGTAT		107.15	0.994	[27]
*Lsi-18S*	ribosome RNA	AGCCTGAGAAACGGCTACCACATC	ACCAGACTCGAAGAGCCCGGTAT		97.636	0.993	[27]
**mRNA**	**Gene description**	**Forward primer (5'-3')**	**Reverse primer (5'-3')**		**Amplication efficiency (%)**	**R**^**2**^	**Reference**
*ClYLS8*	Yellow-leaf-specific proein8	AGAACGGCTTGTGGTCATTC	GAGGCCAACACTTCATCCAT		105.159	0.995	[20]
*CmYLS8*	Yellow-leaf-specific proein8	AGAACGGCTTGTGGTCATTC	GAGGCCAACACTTCATCCAT		99.648	0.994	[20]
*CmPP2A-1*	Protein phosphatase 2A regulatory subunit A	AAGAGCCCACCAGCTTGTAA	TGTTCTCCCCAATCTCAAGG		96.114	0.993	[20]
*LsPP2A-2*	Protein phosphatase 2A regulatory subunit A	TGGTAGCATCCTTTCCCAATACA	CATGCCCGTTCAGCTTTAGC		106.171	0.999	[26]

### Total RNA isolation and cDNA synthesis

Total RNA was extracted using the mirVana^™^ miRNA Isolation Kit (Ambion) in accordance with the instructions provided by manufacturer. RNA quantity and quality was assessed spectrophotometrically (Nano-Drop) and samples showing A260/A280 ratio of 1.8–2.0 as well as A260/A230 ratio of 2.0–2.2 were used for subsequent analysis.

Total RNA from each sample, which included miRNA, was reverse transcribed using an NCode^™^ VILO^™^ miRNA cDNA Synthesis Kit (Invitrogen) in accordance with the manufacturer’s protocol. As per this protocol, the first-strand cDNA was synthesized by reverse transcribing 500 ng of the total RNA using a universal Oligo-dT adaptor primer in a final reaction volume of 20 μl. All cDNA samples were stored at -20°C until further use.

### Quantitative RT-PCR analysis

Quantitative RT-PCR was performed by using the FastStart Universal SYBR Green Master (ROX) kit (Roche) on the StepOne-plus machine (ABI), following the manufacturer’s instructions. The amplification was performed at 95°C for 10 min, 40 cycles at 95°C for 10 s, and 60°C for 30 s. Melt curves analysis was performed immediately after the completion of qRT-PCR to assess non-specific amplification. Amplification efficiency for all primer pairs was evaluated using serial two-fold dilutions of pooled cDNA (400, 200, 100, 50, 25 ng). All assays were replicated three times and no template controls were included.

### Data analysis

Primer efficiency (E) and correlation coefficients (R^2^) were automatically determined for all plates using StepOne v2.3 software. Because of the difference in their genomes, the specificity of the designed primer was analyzed respectively to each species (watermelon, squash, and bottle gourd). The expression stability of each candidate reference gene was analyzed using geNorm [30] and NormFinder [31] software.

The Ct values of three biological replicates were averaged arithmetically and converted into linear relative quantity (RQ) values using the equation RQ = 2^^-(Ct Sample-Ct Calibrator)^. For every gene, the sample with the lowest Ct value was set as a calibrator, and therefore the expression level was equal to one (S3 Table). The gene expression stability value M can be calculated by geNorm, describing the variation of a gene compared to all other candidate genes (S4 Table). It also can determine the optimum number of multiple reference genes by counting the pairwise variation V2/3 and performing a stepwise calculation of the Vn/n+1. V2/3 is the variation between normalizer factors NF2 (the two most stable reference genes) and NF3 (the three most stable reference genes). The variation (V) values should be below the cutoff (0.15) and indicate the optimal number of reference genes for more reliable normalization. In contrast, the NormFinder calculates the expression stability value M for the individual reference gene by using a model-based approach with consideration of variations across groups (S5 Table). For these two statistical methods, the lowest M always suggests the most stable gene.

### Reference gene validation

To confirm the reliability of the potential reference genes, *miR85* (19 nt), a putative nutrient stress-responsive miRNA, was selected from the small RNA-seq experiment. The information of its sequence and normalized reads was illustrated in S8 Table. The expression of *miR85*, was normalized with the most stable and multiple best reference genes (V<0.15, determined by geNorm), in the N and P stress groups, respectively. The least stable reference gene was also used to compare the difference in normalization results. Similarly, the expression of *Cla-miR164a*, *Cmo-miR397a*, and *Lsi-miR5148a* were measured separately, and normalized with five common candidate reference genes in the scion and rootstocks under normal growth conditions, which was subsequently compared with the normalized reads obtained by small RNA-seq experiments. NF was calculated as the geometric mean of the used reference genes. Information on the target genes and their primers is listed in Table 1. Specificity of the primers was determined by the melting curve analysis (S1 Fig). Three biological replicates were adopted for each growth condition and three technical replicates were adopted for each sample. The qRT-PCR amplification conditions were same as those described above. To compute the relative expression levels of all the four target genes, we used the 2^-ΔΔCt^ method as described by Livak et al. [32].

### Statistical analysis

For statistical analyses, one-way ANOVA and the post hoc Tukey test were performed using SPSS Statistics Version 22 (IBM software, Inc.; www-01.ibm.com/software/).

## Results

### Amplification efficiency and specificity of each candidate reference gene

To identify suitable reference genes for expression analysis of miRNA in grafted watermelon scion and the two rootstocks under normal or nutrient starvation conditions, a comprehensive set of seventeen candidate genes was selected. The amplification efficiencies (E%) for all primer pairs were determined by qRT-PCR standard curve assays, since optimal amplification conditions are indispensable for robust and precise quantification of samples. As shown in Table 1, the amplification efficiency for each primer pair varying from 83.609% (*Cla-miR82*) to 114.628% (*Cmo-miR397a*) and the R^2^ values, which represents the relationship between the raw Ct values and the corresponding relative amount of template, were all higher than 0.98. Ideally, the efficiency of a qRT-PCR should be 100%, as the cDNA template doubles at every cycle during the exponential phase [17]. In addition, melting curve analyses were also conducted for every primer pair and a single peak was generated instantly after completion of PCR amplification (S1 Fig). These results indicate that the selected primer pairs are suitable for qRT-PCR of high-efficiency and specificity.

### Expression profiles of the candidate reference genes

**Fig 1:**
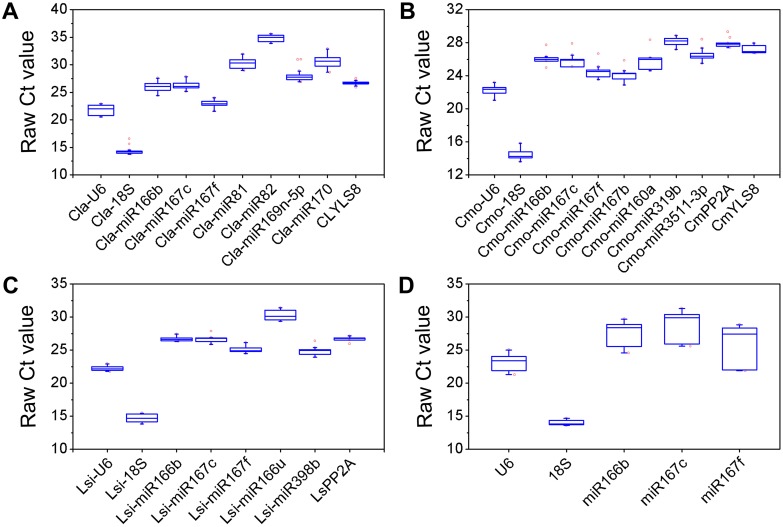
Box plot of the Ct values of the candidate reference genes among samples. The raw Ct values of each reference gene in samples from four datasets: (A) the leaves of different grafted watermelons under N or P starvation, (B) the roots of grafted and non-grafted squash rootstocks under N or P starvation, (C) the roots of grafted and non-grafted bottle gourd rootstocks under N or P starvation, (D) all leaves from different grafted watermelon scions and roots from grafted and non-grafted rootstocks under normal conditions. The line across the *box* depicts the median. The *box* indicates the 25/75 percentiles, and *whisker* caps represent the maximum and minimum values. The circles represent the outliers.

The expression levels of seventeen candidate reference genes were examined by qRT-PCR on samples collected from scion and rootstocks under normal growth, as well as under N and P starvation. The Ct value was normally used to give a preliminary overview of the expression levels of the reference genes [33]. *Cla-miR82*, which had the highest mean Ct value (35.06), was generally expressed at the lowest level among the candidate reference genes in the watermelon scions leaves. By contrast, *Cla-18S* with lowest mean Ct value (14.20), showed the highest transcription level. In addition, *ClYLS8*, *Cla-miR82*, *Cla-miR167f* and *Cla-U6* genes showed a lower variation in expression (lower than 2.50 cycles) under different nutrient conditions, whereas *Cla-miR169-5p* and *Cla-miR170* showed a significant higher variation in expression (higher than 4 cycles) compared with the other reference genes (Fig 1A and S6 Table). In squash roots, the mean Ct values for all of the 11 candidate reference genes ranged from 14.27 (*Cmo-18S*) to 28.30 (*Cmo-miR319b*). Besides, *Cmo-miR160a* showed highest variability of Ct value, whereas *CmYLS8* exhibited the lowest expression variations under different grafting and nutrient conditions. Most of candidate reference genes showed an expression range in Ct values below 3 cycles (Fig 1B and S6 Table). Expression profile analysis was performed on 8 candidate reference genes in bottle gourd roots. Among them, *Lsi-18S* accumulated at the highest level, whereas, *Lsi-miR166u* exhibited the least transcription abundance. Moreover, *Lsi-miR398b* and *Lsi-miR166b* showed the highest and lowest expression variations, respectively, under different grafting and nutrient conditions (Fig 1C and S6 Table). Five common candidate reference genes were selected for further analysis in both scion and rootstocks under normal growth conditions. The genes *18S* and *miR167c* were expressed at the highest and least levels, respectively. Most of the genes showed high expression variations in this analysis (Fig 1D and S6 Table). Considering the variations in the amount of starting materials between the samples as well as operations of qRT-PCR, the expression stability of each reference gene could not be accurately estimated by the direct comparison of their raw Ct values. Therefore, it is necessary to calculate expression variation and select reliable reference genes by more powerful methods.

### Expression stability analysis

**Fig 2:**
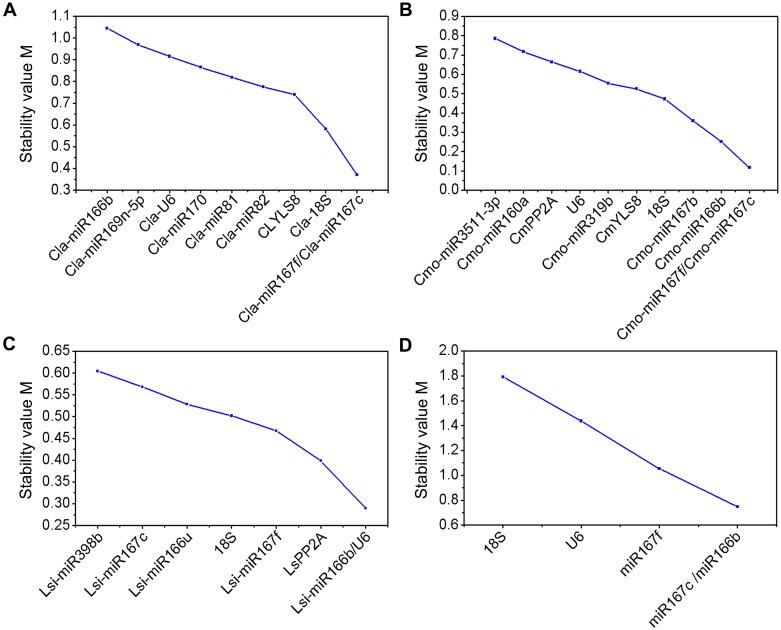
Average expression stability values (M) of the tested candidate reference genes determined by geNorm. Stability value of each reference gene was evaluated from four sample subsets: (A) scion leaves of different grafted watermelons submitted to N or P stress, (B) roots of grafted and non-grafted squash submitted to N or P stress, (C) roots of grafted and non-grafted bottle gourd submitted to N or P stress, (D) all scion leaves of different grafted watermelons and roots of grafted and non-grafted rootstocks under normal conditions. The most stable reference genes were measured during the stepwise exclusion of the least stable reference genes. The lower the M values the more stable expression of candidate reference genes.

**Fig 3:**
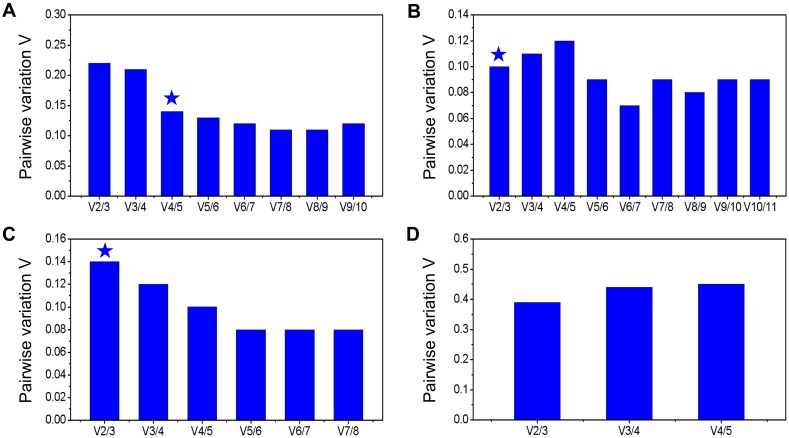
Pairwise variation analyses of candidate reference genes calculated by geNorm. Pairwise variation (V) was calculated by geNorm to determine the optimal number of reference genes required for accurate normalization in different sample sets: (A) scion leaves of different grafted watermelons submitted to N or P stress, (B) roots of grafted and non-grafted squash submitted to N or P stress, (C) roots of grafted and non-grafted bottle gourd submitted to N or P stress, (D) all scion leaves of different grafted watermelons and roots of grafted and non-grafted rootstocks under normal conditions. The Vn/n+1 value was calculated for every comparison between two consecutive candidate reference genes. The inclusion of additional reference genes is not required if the Vn/n+1value less than the recommended 0.15 threshold.

**Fig 4:**
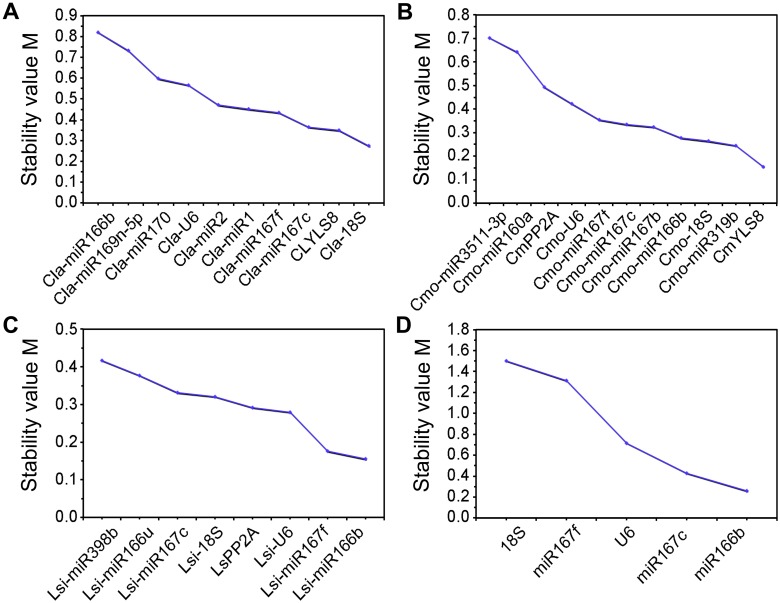
Candidate reference genes ranked according to their expression stability calculated by NormFinder. Average expression stability value of each reference gene was determined from (A) scion leaves of different grafted watermelons submitted to N or P stress, (B) roots of grafted and non-grafted squash submitted to N or P stress, (C) roots of grafted and non-grafted bottle gourd submitted to N or P stress, (D) all scion leaves of different grafted watermelons and roots of grafted and non-grafted rootstocks under normal conditions. The lower the M value, the more stable expression of candidate reference genes.

The geNorm and NormFinder are widely used to determine the stability of reference genes [30, 31]. Here, the samples were divided into four different subsets and the ranks of the selected reference genes were first determined by geNorm and showed in Fig 2 and S4 Table. Most of the candidate reference genes showed acceptable expression stabilities (M ≤ 1.5). When leaf samples from scions under different grafting conditions and nutrition stresses were considered, *Cla-miR167c* and *Cla-miR167f* showed the lowest average expression stability value, whereas *Cla-miR166b* showed the highest M value (Fig 2A). These results suggest that *miR167f* and *miR167c* had the most stable expression in scion, whereas *miR166b* had the highest level of expression variation. In addition, pairwise variation between two sequential normalization factors (NFs) containing an increasing number of reference genes was also calculated by geNorm to determine the optimum number of genes required for a more reliable normalization. The inclusion of additional reference genes is not required if the V value is below 0.15 [30]. The results revealed that the pairwise variation V4/5 (V = 0.142) is the first value that is lower than the 0.15 threshold (Fig 3A and S4 Table). This result suggested that across all the samples, at least four reference genes, including *Cla-miR167f*, *Cla-miR167c*, *Cla-18S* and *ClYLS8*, were required for more reliable normalization of target genes. Similarly, NormFinder identified all of aforementioned genes as stable reference genes (M ≤ 1.5). Among them, *Cla-miR166b* showed the highest stability value, whereas *Cla-18S* was identified as the most stable gene (Fig 4A and S5 Table).

In squash rootstocks subset, *Cmo-miR167c* and *Cmo-miR167f* (homologous to *Cla-miR167c* and *Cla-miR167f*) were identified as the two most stable genes under different grafting conditions and nutrition stresses (Fig 2B). These two genes were also satisfactory for more reliable normalization because the variation value V2/3 (V = 0.104) was below the cutoff value of 0.15 (Fig 3B). Both algorithms disclosed *Cmo-miR3511-3p* as the least stable gene. The *CmYLS8* was identified as the best reference gene by NormFinder (Fig 4B).

In the bottle gourd rootstock subset, both geNorm and NormFinder identified *Lsi-miR166b* and *Lsi-miR398b* as the most and least stable genes, respectively (Figs 2C and 4C). In addition, *Lsi-U6* also exhibited the highest expression stability in geNorm analysis. Moreover, pairwise variation analysis on these 8 reference genes showed that *Lsi-miR166b* and *Lis-U6* were sufficient for more reliable normalization (Fig 3C).

Five common reference genes (*miR167c*, *miR167f*, *miR166b*, *U6*, *18S*) were selected for expression stability analysis in all samples from scion and rootstocks under normal growth condition. The *miR166b* and *miR167c* genes were ranked as the two most stable reference genes for calculating target genes by both algorithms, whereas *18S* was identified as the least stable reference gene (Figs 2D and 4D). However, geNorm pairwise variation analysis showed that all of the V values were higher than 0.15, which indicates that more stable reference genes were necessary for optimal normalization expression analysis in different species and tissues simultaneously (Fig 3D). Taken together, even though most of the candidate reference genes were identified by both geNorm and NormFinder as acceptable reference genes (M ≤ 1.5), m*iR167c*, *miR167f* and *miR166b* were most frequently recommended by both algorithms in different sample subsets.

### Validation of putative reference genes

**Fig 5:**
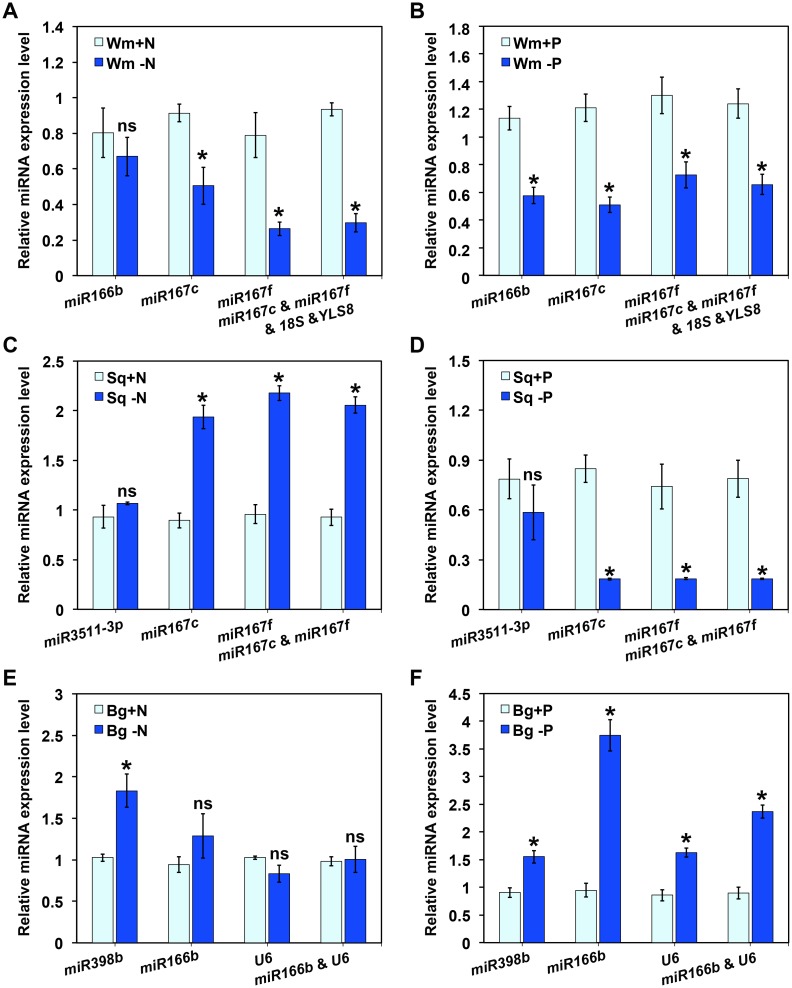
Relative expression of *miR85* in grafted watermelon under nutrient stresses. *miR85* expression levels in (A) and (B) leaves of self-grafted scions, (C) and (D) roots of non-grafted squash, (E) and (F) roots of non-grafted bottle gourd under N or P stresses were normalized by the least stable, single most stable and best pair of reference genes (range from left to right). For the pair of best reference genes and multiple reference genes, their geometric mean was calculated and used for normalization. The relative expression levels are exhibited as the mean ± SD, which was calculated from three biological replicates. Asterisk (*) indicates significant difference and ns indicates no significant difference between +N and –N, +P and -P (*P* < 0.05, one-way ANOVA and then Tukey’s test for multiple comparisons).

To validate the selected reference genes in the nutrient stress group, the relative expression profiles of *miR85*, a potential nutrient-stress-responsible novel miRNA in *Cucurbitaceae*, was normalized by the most stable reference gene and the multiple best reference genes (determined by geNorm), as well as the least stable reference gene (determined by both algorithms). After N starvation, the expression of *miR85* was significantly down-regulated in the watermelon leaves and up-regulated in squash roots under N deficient stress, on normalization by the respective single best reference gene alone and the best pair of reference genes. By contrast, *miR85* showed no significant difference between sufficient and deficient N growth conditions when the least stable reference gene was used for normalization (Fig 5A–5C and S7 Table). In the bottle gourd roots, when the most stable (*miR166b* or *U6*) and best pair of reference genes (*miR166b* and *U6*) were used for normalization, *miR85* showed no significant change between different N conditions. Whereas, the result normalized by the most unstable reference gene (*miR398b*) exhibited significant enhancement by the nitrogen stress (Fig 5E).

In the P starvation groups, the relative expression of *miR85* in watermelon leaves and squash roots was significantly down-regulated by P starvation on normalizing by the respective single best reference gene alone and the best pair of reference genes (Fig 5B–5D); however, no significant response of squash roots to P deficiency could be observed when the most unstable reference gene (*miR3511-3p*) was used (Fig 5D). In the bottle gourd roots, enhanced expression of *miR85* was observed after P starvation notwithstanding the identity of the reference genes used (Fig 5F).

**Fig 6:**
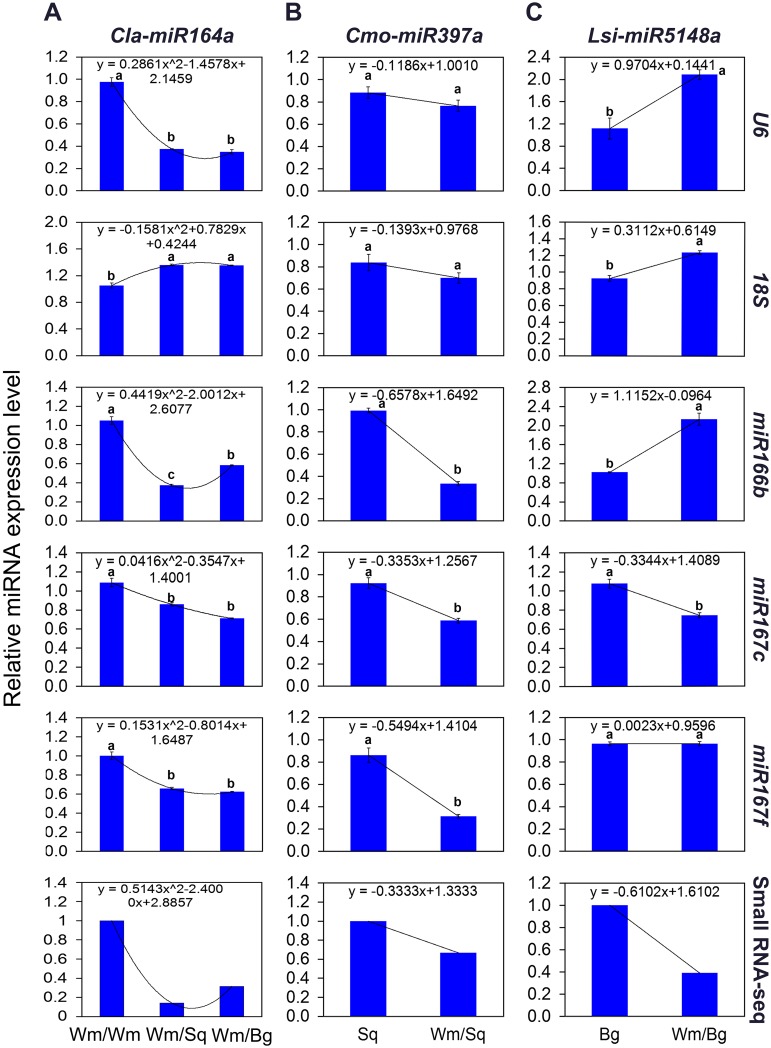
Expression profiles of target miRNAs in grafted watermelon under normal growth conditions. (A) Expression pattern of *Cla-miR164a* in the leaves of grafted watermelon scions. “Wm/Wm”, “Wm/Sq” and “Wm/Bg” represent self-, squash- and bottle gourd-grafted watermelon, respectively. (B) Expression pattern of *Cmo-miR397a* in squash roots. “Sq” and “Wm/Sq” represent non-grafted and grafted squash, respectively. (C) Expression pattern of *Lsi-miR5148a* in bottle gourd roots. “Bg” and “Wm/Bg” represent non-grafted and grafted bottle gourd, respectively. qPCR data was normalized by each reference gene, including *U6*, *18S*, *miR166b*, *miR167c* and *miR167f*, respectively. Different lower-case letters denote significant differences among relative transcript levels (*P* < 0.05, one-way ANOVA and then Tukey’s test for multiple comparisons). Values are means (n = 3) ± SD.

To validate the reference genes recommended by geNorm and NormFinder under normal growth conditions, we evaluated the expression of several target genes, including *Cla-miR164a*, *Cmo-miR397a*, and *Lsi-miR5148a* in watermelon leaves, squash roots, and bottle gourd roots respectively. The relative expression levels of these target genes in qRT-PCR assays were normalized by five candidate reference genes (*miR167f*, *miR167c*, *miR166b*, *U6*, *18S*), individually. In the small RNA-seq assay, *Cla-miR164a* was found to exhibit a significant reduction in expression levels in the squash- and bottle gourd-grafted watermelon leaves compare with those of the self-grafted leaves (S8 Table). In the sequencing assay, *Cla-miR164a* showed the highest and lowest similar expression patterns when it was normalized by *miRl66b* and *18S*, respectively. The other reference genes exhibited normalization results relatively less similar to that of sequencing experiment (Fig 6A and S9 Table). The small RNA-seq analysis determined a significant reduction in the expression levels of *Cmo-miR397a* between squash rootstock and non-grafted squash root (S8 Table). Similar expression patterns were obtained when *Cmo-miR397a* was normalized by most of the reference genes. Among these, *miR167c* is likely the best, as its normalization is extremely close to the expression trend identified by the sequencing experiment (Fig 6B and S9 Table). In bottle gourd roots, the accumulation levels of *Lsi-miR5148a* showed a similar expression pattern to that of the sequencing results when normalized by *miR167c* (S8 Table). By contrast, the expression levels of *Lsi-miR5148a* significantly differed with the small RNA-seq when *U6*, *18S* and *miR166b* were employed as reference genes (Fig 6C and S9 Table). These results indicated that, under normal growth conditions, *miR166b* and *miR167c* were the most appropriate reference genes for miRNA expression normalization in the scion and rootstocks (both squash and bottle gourd), respectively. Moreover, the miRNA expressional response to grafting was often misinterpreted when *18S* was used as the internal control.

## Discussion

Quantitative gene expression analysis is an important and effective method to elucidate gene function and regulatory network in biological researches. Recently, qRT-PCR has emerged as a widely used technique and one of most reliable methods for gene expression analysis [34]. Compared with other conventional methods, such as the northern blot and microarray, qRT-PCR has many distinct advantages such as high sensitivity, high specificity, and a wide quantification range [35–38]. Precise and reliable calculations of qRT-PCR results rely heavily on the use of appropriate reference genes to normalize gene expression and minimize non-biological variation between different samples [39].

Peltier et al.[40] and Timoneda et al.[41] demonstrated that miRNAs are more stable than protein-coding genes and therefore could be used as reference genes for miRNA normalization in humans and porcines. It was also seen in some plants, such as soybean, wheat and longan, that miRNAs have higher expression stability than protein-coding genes and some miRNAs are suitable reference genes in accurate normalization of both miRNA and mRNA [17, 18, 19]. To date, Kong et al. [20] performed the first reference gene selection in watermelon under biotic stresses (*Fusarium wilt* and bacterial fruit blotch), abiotic stresses (low temperature, salinity and drought) and normal growth conditions. Nevertheless, the identification of appropriate reference genes for miRNA normalization in the grafted watermelon (including both scion and rootstocks) under low N, P stresses is thus far lacking. Therefore, it is necessary to evaluate suitable reference genes for normalization of miRNA expression in grafted watermelon plants. To evaluate suitable reference genes for normalization of miRNA expression in grafted watermelon, eight potential miRNA reference genes were selected from small RNA-seq experiments, together with seven putative reference genes from the genes that had been validated in other crops [18, 20, 21, 24–26]. Two other frequently used reference genes [27, 42] were also used for evaluation. The geNorm and NormFinder were used to determine the best suitable reference gene sets for each sample group. Even though differences were found in the ranking orders of putative reference genes, the top four stable genes, as well as the most stable and unstable candidate reference gene were almost similar in the two algorithms for each sample set (Figs 2 and 4). The slight discrepancies may have resulted from the different statistical algorithms models used. Similar inconsistencies between these two applets were also reported in other studies [17, 20–22].

In the present study, *miR167c* or *miR166b* were ranked as the most stable reference genes in all scions and rootstocks under normal conditions by geNorm and NormFinder (Figs 2D and 4D). However, the best reference gene changed accordingly, when samples were subdivided into different subsets based on different species and nutrition stress. geNorm identified *miR167c* and *miR167f* as the most stable genes in the watermelon and squash samples grown under low N and P stresses, whereas *miR166b*, determined by both software, stably expressed in the bottle gourd under nutrient starvation (Figs 2A, 2B, 2C and 4A, 4B, 4C). Interestingly, compare with *miR166b*, *miR166u*, another member of miR166 family, was identified as less stable gene evaluated using two software. Similarly, *miR167* was found to be one most appropriate inner reference genes in wheat exposed to biotic and abiotic stress treatments [18]. Machado et al. [21] also indicated that *miR167-1_2* was one of the most stable reference genes for expression analysis during *Brassica napus* seed development. In soybean, *miR167c* was observed as the most unstable gene under biotic stress [17]. These findings indicated that conserved miRNA family may exhibit diverse expression stabilities in different plant species, as well as under various stress conditions.

*U6* is *a* traditional reference gene for miRNA quantification and its expression was found to be steady in citrus somatic embryonic and adult tissues [16]. In this study, *U6* was recommended by geNorm as one of the best reference genes in bottle gourd root but was ranked among the less stably expressed inner controls in other species (Figs 2 and 4). It was identified as an unsuitable endogenous reference gene for the normalization of circulating miRNAs in different populations’ serum and its expression decreased after cycles of freezing and thawing [43]. The *18S* gene, another commonly used internal control gene, usually exhibited extremely higher expression levels [20, 44, 45]. In some plants, *18S* was regarded as the most stably expressed reference gene [46–50]. However, it was identified to be an unsuitable reference gene for the quantification analysis of resistance and susceptibility in melon genotypes [44]. Furthermore, Kong et al. [20] concluded that *18S* was an unstable reference gene in watermelon under specific growth conditions. We also concluded that *18S* acted as the least stable reference gene in the scion (watermelon) and rootstock (squash and bottle gourd) grown together under normal conditions (Figs 2, 4 and 6). The protein-coding gene *YLS8* is an appropriate reference gene for normalization of gene expression in *Arabidopsis*, watermelon and pear [20, 51, 52]. However, it was found to be a less stable candidate gene under heavy metal stress treatment and different nitrogen nutrition [53, 54]. In this study, *YLS8* generally ranked in the intermediate positions by geNorm, which demonstrated that *YLS8* may not be the best reference gene in studies of grafted watermelon and squash under N, P limitation. *PP2A*, which plays an essential role in regulating growth and development, was ranked as the best reference gene for different abiotic stresses (salt, hormonal, and cold treatment) in Zucchini by both geNorm and NormFinder [26, 55]. It was also found to be one of the most highly stable genes in *Arabidopsis* under abiotic stresses and a suitable gene for bud developmental stages and flowering and in the different genotypes of *Prunus mume* [22, 56]. Here, both geNorm and Normfinder ranked *PP2A* was ranked as relatively stable reference gene for normalizing gene expression in squash and bottle gourd rootstocks subjected to N and P starvation (Figs 2 and 4). These results suggested that all of the aforementioned genes could be suitable reference genes only if they were selected based on the experimental conditions.

A novel miRNA *miR85*, identified in small-RNA seq, was used as a target gene to test the impact of reference genes with different stabilities on normalization. The normalization results of *miR85* in nutrient stress conditions using the best reference alone, and the best multiple reference genes assessed by geNorm showed similar expression patterns, differing significantly from those normalized by the most unstable reference gene (Fig 5). Reference gene validations have also been performed in watermelon scions and rootstocks under normal growth conditions. The expression of target miRNAs showed similar changing patterns to those of the small RNA-seq results, when *miR166b* or *miR167c* were used for normalization, which validated that *miR166b* and *miR167c* were the most stable reference genes in watermelon scion and rootstocks, respectively, under normal growth conditions. In contrast, *18S*, the least stable reference gene determined by both algorithms, produced biases that lead to misinterpreted gene expression (Fig 6). These results were in strong agreement with those of the geNorm and NormFinder analyses (Figs 2 and 4). In bottle gourd, normalization result of *miR166b*, one of the recommended reference genes, significantly differs from RNA-seq data (Fig 6C). This finding suggested that more reliable and sensitive gene expression technique, such as RNA-seq analysis, is valuable for the validation of geNorm and NormFinder results.

In conclusion, despite slight differences found in diverse sample subsets, our results indicated that several miRNAs (*miR167c*, *miR167f* and *miR166b*), which usually show higher stability than protein-coding genes (*YLS8* and *PP2A*); appear to be superior reference genes for miRNA expression normalization in our experimental conditions. Moreover, as a certain level of variation always exists for each reference gene, multiple reliable reference genes are recommended to attain a more precise normalization of gene expression in grafted watermelon. In addition, the identified appropriate reference genes, which showed high stability in both scion and rootstocks under different nutrient stresses, will lead to easier and better normalization of target miRNAs levels in grafted watermelon in the future.

## Supporting Information

S1 Fig Melting curve analyses on the candidate reference and target genes. (TIF)

S1 Table Formulas of nutrient solution. (PDF)

S2 Table Detailed information about the reference genes from small RNA-seq in watermelon, squash, and bottle gourd. (PDF)

S3 Table RQ values for stability (M) and variation values (V) calculation. (PDF)

S4 Table Grafted watermelon reference genes ranked according to their expression stability as determined by geNorm in different sample sets. (PDF)

S5 Table Grafted watermelon reference genes ranked according to their expression stability as determined by NormFinder in different sample sets. (PDF)

S6 Table Raw Ct values of each reference gene among different samples. (PDF)

S7 Table Expression of *miR85* in self-grafted watermelon and non-grafted squash and bottle gourd under nutrient stresses. (PDF)

S8 Table Expression profiles of target miRNAs retrieved from small RNA-seq. (PDF)

S9 Table Relative expression of *Cla-miR164a*, *Cmo-miR397a*, *Lsi-miR5148a*. (PDF)

## References

[1] Theodorou ME, Plaxton WC. Metabolic adaptations of plant respiration to nutritional phosphate deprivation. Plant Physio. 1993; 101: 339–344. 10.1104/pp.101.2.339 PMC160576 12231689

[2] Galloway JN, Cowling EB. Reactive nitrogen and the world: 200 years of change. Ambio. 2002; 31: 64–71. 12078011 10.1579/0044-7447-31.2.64

[3] Elser JJ, Bracken MES, Cleland EE, Gruner DS, Harpole WS, Hillebrand H, et al Global analysis of nitrogen and phosphorus limitation of primary producers in freshwater, marine and terrestrial ecosystems. Ecology Letters. 2007; 10: 1135–1142. 10.1111/j.1461-0248.2007.01113.x 17922835

[4] McAllister CH, Beatty PH, Good AG. Engineering nitrogen use efficient crop plants: the current status. Plant Biotechnol. J. 2012; 10: 1011–1125. 10.1111/j.1467-7652.2012.00700.x 22607381

[5] Xu GH, Fan XR, Miller AJ. Plant nitrogen assimilation and use efficiency. Annu. Rev. Plant Biol. 2012; 63: 153–182. 10.1146/annurev-arplant-042811-105532 22224450

[6] Liu N, Yang JH, Guo SG, Xu Y, Zhang MF. Genome-wide identification and comparative analysis of conserved and novel microRNAs in grafted watermelon by high-throughput sequencing. PLoS ONE. 2013; 8: e57359 10.1371/journal.pone.0057359 23468976 PMC3582568

[7] Davis AR, Perkins-Veazie P, Sakata Y, López-Galarzac S, Maroto JV, Lee S-G, et al Cucurbit grafting. Crit. Rev. Plant Sci. 2008; 27: 50–74.

[8] Tateishi K. Grafting watermelon onto pumpkin. J. Jpn. Hortic. 1927; 39: 5–8. (In Japanese).

[9] Sato N, Takamatsu T. Grafting culture of watermelon. Nogyo sekai. 1930; 25: 24–28. (in Japanese).

[10] Fujii H, Chiou T-J, Lin S-I, Aung K, Zhu JK. A miRNA involved in phosphate-starvation response in Arabidopsis. Curr. Biol. 2005; 15: 2038–2043. 10.1016/j.cub.2005.10.016 16303564

[11] Chiou T-J, Aung K, Lin S-L, Wu C-C, Chiang S-F, Su C-L. Regulation of phosphate homeostasis by microRNA in Arabidopsis. Plant Cell. 2006; 18: 412–421. 10.1105/tpc.105.038943 16387831 PMC1356548

[12] Hu B, Zhu CG, Li F, Tang JY, Wang YQ, Lin A, et al LEAF TIP NECROSIS1 plays a pivotal role in the regulation of multiple phosphate starvation responses in rice. Plant Physiol. 2011; 156: 1101–1115. 10.1104/pp.110.170209 21317339 PMC3135962

[13] Zhao M, Ding H, Zhu J-K, Zhang FS, Li W-X. Involvement of miR169 in the nitrogen-starvation responses in Arabidopsis. New Phytol. 2011; 190: 906–915. 10.1111/j.1469-8137.2011.03647.x 21348874 PMC3586203

[14] Baek D, Kim MC, Chun HJ, Kang S, Park HC, Shin G, et al Regulation of miR399f transcription by AtMYB2 affects phosphate starvation responses in Arabidopsis. Plant Physiol. 2013; 161: 362–373. 10.1104/pp.112.205922 23154535 PMC3532267

[15] Pant BD, Buhtz A, Kehr J, Scheible W-R. MicroRNA399 is a long-distance signal for the regulation of plant phosphate homeostasis. The Plant Journal. 2008; 53: 731–738. 10.1111/j.1365-313X.2007.03363.x 17988220 PMC2268993

[16] Kou S-J, Wu X-M, Liu Z, Liu Y-L, Xu Q, Guo W-W. Selection and validation of suitable reference genes for miRNA expression normalization by quantitative RT-PCR in citrus somatic embryogenic and adult tissues. Plant Cell Rep. 2012; 31: 2151–2163. 10.1007/s00299-012-1325-x 22865195

[17] Kulcheski FR, Marcelino-Guimaraes FC, Nepomuceno AL, Abdelnoor RV, Margis R. The use of microRNAs as reference genes for quantitative polymerase chain reaction in soybean. Anal. Chem. 2010; 406: 185–192. 10.1016/j.ab.2010.07.020 20670612

[18] Feng H, Huang XL, Zhang Q, Wei GR, Wang XJ, Kang ZS. Selection of suitable inner reference genes for relative quantification expression of microRNA in wheat. Plant Physiol. Biochem. 2011; 51: 116–122. 10.1016/j.plaphy.2011.10.010 22153247

[19] Lin YL, Lai ZX. Evaluation of suitable reference genes for normalization of microRNA expression by real-time reverse transcription PCR analysis during longan somatic embryogenesis. Plant Physiol. Biochem. 2013; 66: 20–25. 10.1016/j.plaphy.2013.02.002 23454294

[20] Kong QS, Yuan JX, Gao LY, Zhao S, Jiang W, Huang Y, et al Identification of suitable reference genes for gene expression normalization in qRT-PCR analysis in watermelon. PLoS ONE. 2014; 9: e90612 10.1371/journal.pone.0090612 24587403 PMC3938773

[21] Machado RD, Christoff AP, Loss-Morais G, Margis-Pinheiro M, Margis R, Körbes AP. Comprehensive selection of reference genes for quantitative gene expression analysis during seed development in *Brassica napus*. Plant Cell Rep. 2015; 34: 1139–1149. 10.1007/s00299-015-1773-1 25721200

[22] Wang T, Lu JX, Xu ZD, Yang WR, Wang J, Cheng TR, et al Selection of suitable reference genes for miRNA expression normalization by qRT-PCR during flower development and different genotypes of *Prunus mume*. Scientia Horticulturae. 2014; 169: 130–137.

[23] Hoagland DR, Arnon DI. The water culture method for growing plants without soil. Circular California Agricultural Experiment Station. 1950; 347(5406): 357–359.

[24] Borowski JM, Galli V, Messias Rda S, Perin EC, Buss JH, dos Anjos e Silva SD, et al Selection of candidate reference genes for real-time PCR studies in lettuce under abiotic stresses. Planta. 2014; 239: 1187–1200. 10.1007/s00425-014-2041-2 24573225

[25] Gifford ML, Dean A, Gutierrez RA, Coruzzi GM, Birnbaum KD. Cell-specific nitrogen responses mediate developmental plasticity. PNAS. 2008; 105: 803–808. 10.1073/pnas.0709559105 18180456 PMC2206617

[26] Orebro A, Die JV, Román B, Gómez P, Nadal S, González-Verdejo CI. Selection of reference genes for gene expression studies in zucchini (*Cucurbita pepo*) using qPCR. J. Agric. Food. Chem. 2011; 59: 5402–5411. 10.1021/jf200689r 21476515

[27] Guo SG, Liu JG, Zheng Y, Huang MY, Zhang HY, Gong GY, et al Characterization of transcriptome dynamics during watermelon fruit development: sequencing, assembly, annotation and gene expression profiles. BMC Genomics. 2011; 12: 454 10.1186/1471-2164-12-454 21936920 PMC3197533

[28] Wang TZ, Chen L, Zhao MG, Tian QY, Zhang W-H. Identification of drought-responsive microRNAs in *Medicago truncatula* by genome-wide high-throughput sequencing. BMC Genomics. 2011; 12: 367 10.1186/1471-2164-12-367 21762498 PMC3160423

[29] Mao WH, Li ZY, Xia XJ, Li YD, Yu JQ. A combined approach of high-throughput sequencing and degradome analysis reveals tissue specific expression of microRNAs and their targets in cucumber. PLoS ONE. 2012; 7: e33040 10.1371/journal.pone.0033040 22479356 PMC3316546

[30] Vandesompele J, De Preter K, Pattyn F, Poppe B, Van Roy N, De Paepe A, et al Accurate normalization of real-time quantitative RT-PCR data by geometric averaging of multiple internal control genes. Genome Biol. 2002; 3: 1–11. 10.1186/gb-2002-3-7-research0034 PMC126239 12184808

[31] Andersen CL, Jensen JL, Ørntoft TF. Normalization of real-time quantitative reverse transcription-PCR data: a model-based variance estimation approach to identify genes suited for normalization, applied to bladder and colon cancer data sets. Cancer Res. 2004; 64: 5245–5250. 10.1158/0008-5472.CAN-04-0496 15289330

[32] Livak KJ, Schmittgen TD. Analysis of relative gene expression data using real-time quantitative PCR and the 2(-Delta Delta C (T)). Methods. 2001; 25: 402–408. 10.1006/meth.2001.1262 11846609

[33] Toegel S, Huang W, Piana C, Unger FM, Wirth M, Goldring MB, et al Selection of reliable reference genes for qPCR studies on chondroprotective action. BMC Mol. Biol. 2007; 8: 13 10.1186/1471-2199-8-13 17324259 PMC1820791

[34] Bustin SA. Absolute quantification of mRNA using real-time reverse transcription polymerase chain reaction assays. J. Mol. Endocrinol. 2000; 25: 169–193. 11013345 10.1677/jme.0.0250169

[35] Ginzinger DG. Gene quantification using real-time quantitative PCR: an emerging technology hits the mainstream. Exp. Hematol. 2002; 30: 503–512. 12063017 10.1016/s0301-472x(02)00806-8

[36] Bustin SA, Nolan T. Pitfalls of quantitative real-time reverse-transcription polymerase chain reaction. J. Biomol. Tech. 2004; 15: 155–166. 15331581 PMC2291693

[37] Gahon C, Mingam A, Charrier B. Real-time PCR: what relevance to plant studies? J. Exp. Bot. 2004; 55: 1445–1454. 10.1093/jxb/erh181 15208338

[38] Bustin SA, Benes V, Nolan T, Pfaffl MW. Quantitative real-time RT-PCR—a perspective. J. Mol. Endocrinol. 2005; 34: 597–601. 10.1677/jme.1.01755 15956331

[39] Kong W, Zhao J-J, He L, Cheng JQ. Strategies for profiling microRNA expression. J. Cell. Physiol. 2009; 218: 22–25. 10.1002/jcp.21577 18767038

[40] Peltier HJ, Latham GJ. Normalization of microRNA expression levels in quantitative RT-PCR assays: identification of suitable reference RNA targets in normal and cancerous human solid tissues. RNA. 2008; 14:844–852. 10.1261/rna.939908 18375788 PMC2327352

[41] Timoneda O, Balcells I, Córdoba S, Sanchez A. Determination of reference microRNAs for relative quantification in porcine tissues. PLoS One 2012; 7:e44413 10.1371/journal.pone.0044413 22970213 PMC3438195

[42] Hu J, Sun L, Zhu Z, Zheng Y, Xiong W, Ding Y. Characterization of conserved microRNAs from five different cucurbit species using computational and experimental analysis. Biochimie. 2014; 102: 137–144. 10.1016/j.biochi.2014.03.002 24657600

[43] Xiang M, Zeng Y, Yang R, Xu H, Chen Z, Zhong J, et al U6 is not a suitable endogenous control for the quantification of circulating microRNAs. Biochem. Biophys. Res. Commun. 2014; 454: 210–214. 10.1016/j.bbrc.2014.10.064 25450382

[44] Sestili S, Sebastiani MS, Belisario A, Ficcadenti N. Reference gene selection for gene expression analysis in melon infected by Fusarium oxysporum f.sp. melonis. J. Plant Biochem. Biotechnol. 2013; 3: 1–11.

[45] Wan H, Zhao Z, Qian C, Sui Y, Malik AA, Chen J. Selection of appropriate reference genes for gene expression studies by quantitative real-time polymerase chain reaction in cucumber. Anal. Biochem. 2010; 399: 257–261. 10.1016/j.ab.2009.12.008 20005862

[46] Jarošová J, Kundu JK. Validation of reference genes as internal control for studying viral infections in cereals by quantitative real-time RT-PCR. BMC Plant Biol. 2010; 10: 146 10.1186/1471-2229-10-146 20630112 PMC3095291

[47] Kim BR, Nam HY, Kim SU, Kim SI, Chang YJ. Normalization of reverse transcription quantitative-PCR with house-keeping genes in rice. Biotechnol. Lett. 2003; 25: 1869–1872. 14677714 10.1023/a:1026298032009

[48] Maroufi A, Van Bockstaele E, De Loose M. Validation of reference genes for gene expression analysis in chicory (*Cichorium intybus*) using quantitative real-time PCR. BMC Mol. Biol. 2010; 11: 15 10.1186/1471-2199-11-15 20156357 PMC2830926

[49] Sun ZB, Li SD, Sun MH. Selection of reliable reference genes for gene expression studies in Clonostachys rosea 67–1 under sclerotial induction. J. Microbiol. Methods. 2015; 114: 62–65. 10.1016/j.mimet.2015.05.009 25960431

[50] Zarivi O, Cesare P, Ragnelli AM, Aimola P, Leonardi M, Bonfigli A, et al Validation of reference genes for quantitative real-time PCR in Périgord black truffle (*Tuber melanosporum*) developmental stages. Phytochemistry. 2015; 116: 78–86. 10.1016/j.phytochem.2015.02.024 25778998

[51] Remans T, Smeets K, Opdenakker K, Mathijsen D, Vangronsveld J, Cuypers A. Normalization of real-time RT-PCR gene expression measurements in Arabidopsis thaliana exposed to increased metal concentrations. Planta. 2008; 227: 1343–1349. 10.1007/s00425-008-0706-4 18273637

[52] Xu YY, Li H, Li XG, Lin J, Wang ZH, Yang QS, et al Systematic selection and validation of appropriate reference genes for gene expression studies by quantitative real-time PCR in pear. Acta Physiol. Plant. 2015; 37: 40.

[53] Migocka M, Papierniak A. Identification of suitable reference genes for studying gene expression in cucumber plants subjected to abiotic stress and growth regulators. Mol. Breed. 2011; 28: 343–357.

[54] Warzybok A, Migocka M. Reliable Reference Genes for Normalization of Gene Expression in Cucumber Grown under Different Nitrogen Nutrition. PLoS ONE. 2013; 8: e72887 10.1371/journal.pone.0072887 24058446 PMC3772881

[55] Ahn CS, Han JA, Lee HS, Lee S, Pai HS. The PP2A regulatory subunit Tap46, a component of the TOR signaling pathway, modulates growth and metabolism in plants. Plant Cell. 2011; 23: 185–209. 10.1105/tpc.110.074005 21216945 PMC3051261

[56] Czechowski T, Stitt M, Altmann T, Udvardi MK, Scheible WR. Genome-wide identification and testing of superior reference genes for transcript normalization in Arabidopsis. Plant Physiol. 2005; 139: 5–17. 10.1104/pp.105.063743 16166256 PMC1203353

