# Supplementary material for: Identification of Appropriate Reference Genes for Normalization of miRNA Expression in Grafted Watermelon Plants under Different Nutrient Stresses
Source: PLoS One. 2016 Oct 17;11(10):e0164725. doi: 10.1371/journal.pone.0164725 (PMC5066974; doi:10.1371/journal.pone.0164725)
Supplement: S1 Table — (PDF) [file pone.0164725.s002.pdf]

S1 Table. Formulas of nutrient solution.

N sufficient		N deficient	
Component	Concentration	Component	Concentration
$Ca(NO_3)_2·4H_2O$	2.5mM	$Ca(NO_3)_2·4H_2O$	0.25mM
KNO_3	2.5mM	KNO_3	0.25mM
KH_2PO_4	0.5mM	CaCl2	2.25mM
$MgSO_4·7\ H_2O$	1.4mM	KCl	2.25mM
H_3BO_3	50uM	KH_2PO_4	0.5mM
$MnSO_4.H_2O$	10μm	$MgSO_4·7\ H_2O$	1.4mM
$ZnSO_4.7H_2O$	0.76μm	H_3BO_3	50uM
$CuSO_4.5H_2O$	032μm	$MnSO_4.H_2O$	10μm
$(NH_4)_6Mo_7O_{24}.4H_2O$	0.016μm	$ZnSO_4.7H_2O$	0.76μm
Na2Fe-EDTA	0.077μm	$CuSO_4.5H_2O$	032μm
		$(NH_4)_6Mo_7O_{24}.4H_2O$	0.016μm
		Na2Fe-EDTA	0.077μm

P sufficient		P deficient	
Component	Concentration	Component	Concentration
$Ca(NO_3)_2·4H_2O$	2mM	$Ca(NO_3)_2·4H_2O$	2mM
NH4NO3	0.5mM	NH4NO3	0.5mM
KNO_3	2.5mM	KNO_3	2.5mM
KH_2PO_4	0.5mM	KH_2PO_4	0.01mM
$MgSO_4·7\ H_2O$	1.4mM	K_2SO_4	0.26mM
H_3BO_3	50uM	$MgSO_4·7\ H_2O$	1.4mM
$MnSO_4.H_2O$	10μm	H_3BO_3	50uM
$ZnSO_4.7H_2O$	0.76μm	$MnSO_4.H_2O$	10μm
$CuSO_4.5H_2O$	032μm	$ZnSO_4.7H_2O$	0.76μm
$(NH_4)_6Mo_7O_{24}.4H_2O$	0.016μm	$CuSO_4.5H_2O$	032μm
Na2Fe-EDTA	0.077μm	$(NH_4)_6Mo_7O_{24}.4H_2O$	0.016μm
		Na2Fe-EDTA	0.077μm

All of these formulas were modified from Hoagland solution [23].
