# Supplementary material for: Identification of Appropriate Reference Genes for Normalization of miRNA Expression in Grafted Watermelon Plants under Different Nutrient Stresses
Source: PLoS One. 2016 Oct 17;11(10):e0164725. doi: 10.1371/journal.pone.0164725 (PMC5066974; doi:10.1371/journal.pone.0164725)
Supplement: S2 Table — (PDF) [file pone.0164725.s003.pdf]

S2 Table. Detailed information about the reference genes from small RNA-seq data in watermelon, squash and bottle gourd.

Name[a]	ID	Length (nt)	Sequence	Normalized reads Wm/Wm	Wm/Bg	Wm/Sq	fold-change(log2 (Wm/Bg)/(Wm/Wm))	fold-change(log2 (Wm/Sq)/(Wm/Wm))
Cla-miR81	unconservative_Chr1_116693	24	ATGTCTATCTGGGTCTATCGCAGT	51	62	58	0.281770968	0.185555653
Cla-miR82	unconservative_Chr10_2427841	24	ATTGTTGTTACATAAAGGACGAGT	64	70	77	0.129283017	0.266786541
Cla-miR170	conservative_Chr11_2887302	21	TGATTGAGCCGCGCCAATATC	53	114	90	1.10496956	0.763932642
Cla-miR166b	conservative_Chr6_1495765	21	TCGGACCAGGCTTCATTCCCC	20476	6228	15207	-1.717093062	-0.429198342

Name[b]	ID	Length (nt)	Sequence	Normalized reads Sq	Wm/Sq	fold-change(log2 (Wm/Sq)/Sq)
Cmo-miR3511-3p	conservative_Chr5_1210843	22	AGTTACTAATTAATGATCTGGC	87	80	-0.121015401
Cmo-miR166b	conservative_Chr6_1495765	21	TCGGACCAGGCTTCATTCCCC	11956	13158	0.138205416
Cmo-miR319b	conservative_Chr5_1343674	20	TTGGACTGAAGGGAGCTCCC	13277	17025	0.358725598

Name[c]	ID	Length (nt)	Sequence	Normalized reads Bg	Wm/Bg	fold change(log2 (Wm/Bg)/Bg)
Lsi-miR398b	conservative_Chr3_823696	21	TGTGTTCTCAGGTCGCCCCTA	800	476	-0.749038426
Lsi-miR166u	conservative_Chr6_1516438	21	TCTCGGACCAGGCTTCATTCT	5123	2651	-0.950452222

a, *Citrullus lanatus* miRNA; b, *Cucurbita moschata* miRNA; c, *Lagenaria siceraria* miRNA
