# Supplementary material for: Identification of Appropriate Reference Genes for Normalization of miRNA Expression in Grafted Watermelon Plants under Different Nutrient Stresses
Source: PLoS One. 2016 Oct 17;11(10):e0164725. doi: 10.1371/journal.pone.0164725 (PMC5066974; doi:10.1371/journal.pone.0164725)
Supplement: S3 Table — (PDF) [file pone.0164725.s004.pdf]

S3 Table. RQ values for stability (M) and variation values (V) calculation.

Sample \ Reference gene	*Cla-miR81*	*Cla-miR82*	*Cla-miR167f*	*Cla-U6*	*CLYLS8*	*Cla-18S*	*Cla-miR166b*	*Cla-miR169n-5p*	*Cla-miR170*	*Cla-miR167c*
**N sufficient**										
(Wm/Wm)L	1.00	0.62	0.40	0.49	0.86	0.74	0.49	0.50	0.79	0.62
(Wm/Sq)L	0.17	0.30	0.36	0.87	0.53	0.87	0.31	0.60	0.14	0.47
(Wm/Bg)L	0.38	0.35	0.44	1.00	0.56	0.59	0.29	0.49	0.22	0.35
**N deficient**										
(Wm/Wm)L	0.81	0.84	0.23	0.53	1.00	0.80	0.41	0.56	1.00	0.35
(Wm/Sq)L	0.33	0.80	0.40	0.22	0.57	0.90	0.11	0.92	0.25	0.53
(Wm/Bg)L	0.13	0.33	0.22	0.23	0.57	0.14	0.28	0.06	0.07	0.15
**P sufficient**										
(Wm/Wm)L	0.45	1.00	0.29	0.23	0.62	1.00	0.18	1.00	0.23	0.34
(Wm/Sq)L	0.31	0.46	0.73	0.86	0.66	0.87	0.59	0.60	0.30	1.00
(Wm/Bg)L	0.53	0.37	0.48	0.35	0.56	0.74	0.52	0.42	0.46	0.62
**P deficient**										
(Wm/Wm)L	0.26	0.76	0.48	0.19	0.52	0.71	0.22	0.76	0.16	0.61
(Wm/Sq)L	0.77	0.38	1.00	0.37	0.33	0.68	0.18	0.25	0.40	0.94
(Wm/Bg)L	0.13	0.42	0.19	0.25	0.41	0.28	1.00	0.06	0.05	0.17

Sample \ Reference gene	*Cmo-U6*	*CmYLS8*	*Cmo-18S*	*CmPP2A*	*Cmo-miR167f*	*Cmo-miR3511-3p*	*Cmo-miR167b*	*Cmo-miR166b*	*Cmo-miR160a*	*Cmo-miR167c*	*Cmo-miR319b*
**N sufficient**											
(Sq)R	0.23	0.51	0.44	0.43	0.49	0.28	0.37	0.53	0.41	0.56	0.35
(Wm/Sq)R	1.00	1.00	0.65	0.72	1.00	0.43	0.56	1.00	1.00	1.00	1.00
**N deficient**											
(Sq)R	0.34	0.77	1.00	0.74	0.68	1.00	1.00	0.46	0.34	0.72	0.65
(Wm/Sq)R	0.37	0.87	0.61	0.78	0.45	0.54	0.38	0.42	0.36	0.54	0.62
**P sufficient**											
(Sq)R	0.44	0.88	0.65	0.90	0.34	0.58	0.31	0.38	0.33	0.37	0.43
(Wm/Sq)R	0.40	0.44	0.21	0.69	0.11	0.62	0.13	0.15	0.08	0.14	0.31
**P deficient**											
(Sq)R	0.56	0.96	0.71	1.00	0.79	0.44	0.61	0.57	0.39	1.00	0.39
(Wm/Sq)R	0.25	0.53	0.27	0.26	0.51	0.13	0.39	0.49	0.90	0.57	0.49

Sample \ Reference gene	*Lsi-miR167c*	*Lsi-miR398b*	*Lsi-miR166u*	*Lsi-miR166b*	*Lsi-U6*	*Lsi-miR167f*	*LsPP2A*	*Lsi-18S*
**N sufficient**								
(Bg)R	1.00	1.00	0.32	0.56	0.59	0.56	0.52	0.56
(Wm/Bg)R	0.75	0.50	0.76	0.93	0.60	0.82	0.57	0.79
**N deficient**								
(Bg)R	0.54	0.37	0.25	0.69	0.77	0.35	0.54	0.33
(Wm/Bg)R	0.52	0.47	0.61	0.77	0.87	0.78	0.68	1.00
**P sufficient**								
(Bg)R	0.55	0.46	0.87	1.00	0.83	0.74	1.00	0.81
(Wm/Bg)R	0.49	0.74	1.00	0.80	0.64	1.00	0.61	0.52
**P deficient**								
(Bg)R	0.54	0.68	0.60	0.93	1.00	0.54	0.44	0.33
(Wm/Bg)R	0.26	0.18	0.33	0.47	0.45	0.31	0.56	0.35

Sample \ Reference gene	*miR167c*	*miR166b*	*miR167f*	*18S*	*U6*
(Wm/Wm)L	0.48	0.25	0.51	0.84	0.20
(Wm/Sq)L	0.79	1.00	0.94	0.87	1.00
(Wm/Bg)L	1.00	0.50	1.00	0.47	0.67
(Sq)R	0.05	0.05	0.01	0.93	0.15
(Wm/Sq)R	0.04	0.07	0.01	0.78	0.07
(Bg)R	0.02	0.03	0.01	0.59	0.23
(Wm/Bg)R	0.05	0.06	0.02	1.00	0.28

Wm/Wm: self-grafted watermelon; Wm/Sq: squash-grafted watermelon; Wm/Bg: bottle gourd-grafted watermelon, Sq: non-grafted squash; Bg: non-grafted bottle gourd. "L" represents leaf, "R' represents root.

RQ = 2^-(Ct Sample-Ct Calibrator).
