# Supplementary material for: Identification of Appropriate Reference Genes for Normalization of miRNA Expression in Grafted Watermelon Plants under Different Nutrient Stresses
Source: PLoS One. 2016 Oct 17;11(10):e0164725. doi: 10.1371/journal.pone.0164725 (PMC5066974; doi:10.1371/journal.pone.0164725)
Supplement: S4 Table — (PDF) [file pone.0164725.s005.pdf]

S4 Table. Grafted watermelon reference genes ranked according to their expression stability as determined by geNorm in different sample sets.

Scion samples under N, P starvation	Stability value (M)	Squash rootstock samples under N, P starvation	Stability value (M)	Bottle gourd rootstocks samples under N, P starvation	Stability value (M)	All scion and rootstocks under normal growth conditions	Stability value (M)
*Cla-miR167c**	0.371	*Cmo-miR167c**	0.118	*Lsi-miR166b**	0.290	*miR167c*	0.747
*Cla-miR167f**	0.371	*Cmo-miR167f**	0.118	*Lsi-U6**	0.290	*miR166b*	0.747
*Cla-18S**	0.582	*Cmo-miR166b*	0.251	*LsPP2A*	0.399	*miR167f*	1.053
*CLYLS8**	0.740	*Cmo-miR167b*	0.360	*Lsi-miR167f*	0.468	*U6*	1.437
Cla-miR82	0.776	*Cmo-18S*	0.473	*Lsi-18S*	0.502	*18S*	1.791
Cla-miR81	0.819	*CmYLS8*	0.525	*Lsi-miR166u*	0.528		
Cla-miR170	0.866	*Cmo-miR319b*	0.554	*Lsi-miR167c*	0.568		
Cla-U6	0.916	*Cmo-U6*	0.615	*Lsi-miR398b*	0.605		
Cla-miR169n-5p	0.969	*CmPP2A*	0.664				
Cla-miR166b	1.045	*Cmo-miR160a*	0.717				
		Cmo-miR3511-3p	0.786				

"*": represents the optimal reference genes determined by pairwise variation analyses which are presented in Fig 3. The recommended threshold of 0.15 is adopted.
