# Supplementary material for: Identification of Appropriate Reference Genes for Normalization of miRNA Expression in Grafted Watermelon Plants under Different Nutrient Stresses
Source: PLoS One. 2016 Oct 17;11(10):e0164725. doi: 10.1371/journal.pone.0164725 (PMC5066974; doi:10.1371/journal.pone.0164725)
Supplement: S5 Table — (PDF) [file pone.0164725.s006.pdf]

S5 Table. Grafted watermelon reference genes ranked according to their expression stability as determined by NormFinder in different sample sets.

Scion samples under N, P starvation	Stability value (M)	Squash rootstock samples under N, P starvation	Stability value (M)	Bottle gourd rootstocks samples under N, P starvation	Stability value (M)	All scion and rootstocks under normal growth conditions	Stability value (M)
Cla-18S	0.273	*CmYLS8*	0.155	*Lsi-miR166b*	0.155	*miR166b*	0.259
CLYLS8	0.349	*Cmo-miR319a*	0.245	*Lsi-miR167f*	0.176	*miR167c*	0.429
Cla-miR167c	0.364	*Cmo-18S*	0.263	*Lsi-U6*	0.280	*U6*	0.716
Cla-miR167f	0.434	*Cmo-miR166b*	0.277	*LsPP2A*	0.292	*miR167f*	1.313
Cla-miR1	0.451	*Cmo-miR167b*	0.324	*Lsi-18S*	0.321	*18S*	1.503
Cla-miR2	0.470	*Cmo-miR167c*	0.335	*Lsi-miR167c*	0.332		
Cla-U6	0.566	*Cmo-miR167f*	0.355	*Lsi-miR166h-3p*	0.377		
Cla-miR170	0.597	*Cmo-U6*	0.422	*Lsi-miR398b*	0.417		
Cla-miR169n-5p	0.732	*CmPP2A*	0.492				
Cla-miR166b	0.819	*Cmo-miR160a*	0.642				
		Cmo-miR3511-3p	0.703				
