# Supplementary material for: Identification of Appropriate Reference Genes for Normalization of miRNA Expression in Grafted Watermelon Plants under Different Nutrient Stresses
Source: PLoS One. 2016 Oct 17;11(10):e0164725. doi: 10.1371/journal.pone.0164725 (PMC5066974; doi:10.1371/journal.pone.0164725)
Supplement: S6 Table — (PDF) [file pone.0164725.s007.pdf]

S6 Table. Raw Ct values of each reference gene among different samples.

Sample \ Reference gene	*Cla-U6*	*Cla-18S*	*Cla-miR166b*	*Cla-miR167c*	*Cla-miR167f*	*Cla-miR81*	*Cla-miR82*	*Cla-miR169n-5p*	*Cla-miR170*	*CLYLS8*
**N sufficient**										
(Wm/Wm)L	21.59	14.20	25.44	25.86	22.92	28.97	34.57	27.89	28.97	26.14
(Wm/Sq)L	20.75	13.97	26.09	26.26	23.07	31.54	35.61	27.65	31.50	26.82
(Wm/Bg)L	20.55	14.53	26.20	26.68	22.78	30.36	35.40	27.92	30.85	26.76
**N deficient**										
(Wm/Wm)L	21.45	14.10	25.69	26.67	23.70	29.27	34.14	27.74	28.64	25.91
(Wm/Sq)L	22.71	13.92	27.59	26.07	22.91	30.57	34.21	27.02	30.64	26.72
(Wm/Bg)L	22.66	16.58	26.22	27.85	23.78	31.94	35.47	30.98	32.51	26.73
**P sufficient**										
(Wm/Wm)L	22.66	13.77	26.90	26.73	23.37	30.12	33.88	26.90	30.74	26.60
(Wm/Sq)L	20.77	13.96	25.15	25.16	22.03	30.67	35.00	27.63	30.39	26.50
(Wm/Bg)L	22.04	14.19	25.33	25.84	22.65	29.89	35.33	28.17	29.75	26.74
**P deficient**										
(Wm/Wm)L	22.91	14.27	26.57	25.87	22.65	30.91	34.28	27.30	31.28	26.84
(Wm/Sq)L	21.98	14.33	26.87	25.25	21.58	29.34	35.28	28.88	29.94	27.51
(Wm/Bg)L	22.56	15.62	24.40	27.73	24.00	31.91	35.13	30.93	32.87	27.18
**max-min**	2.37	2.81	3.20	2.69	2.42	2.97	1.73	4.08	4.23	1.60
**median**	22.01	14.20	26.14	26.16	22.91	30.46	35.06	27.82	30.69	26.73

Sample \ Reference gene	*Cmo-U6*	*Cmo-18S*	*Cmo-miR166b*	*Cmo-miR167c*	*Cmo-miR167f*	*Cmo-miR167b*	*Cmo-miR160a*	*Cmo-miR319b*	*Cmo-miR3511-3p*	*CmPP2A*	*CmYLS8*
**N sufficient**											
(Sq)R	23.20	14.80	25.88	25.93	24.56	24.32	25.89	28.68	27.35	28.62	27.73
(Wm/Sq)R	21.06	14.23	24.96	25.09	23.54	23.72	24.62	27.17	26.70	27.87	26.75
**N deficient**											
(Sq)R	22.60	13.61	26.06	25.56	24.11	22.89	26.17	27.80	25.49	27.84	27.13
(Wm/Sq)R	22.50	14.32	26.22	25.97	24.71	24.27	26.09	27.87	26.38	27.77	26.95
**P sufficient**											
(Sq)R	22.25	14.23	26.34	26.52	25.09	24.59	26.20	28.40	26.28	27.56	26.94
(Wm/Sq)R	22.39	15.83	27.72	27.91	26.67	25.86	28.34	28.88	26.17	27.94	27.94
**P deficient**											
(Sq)R	21.89	14.09	25.78	25.09	23.89	23.61	25.99	28.52	26.67	27.41	26.81
(Wm/Sq)R	23.06	15.52	25.99	25.89	24.52	24.26	24.78	28.21	28.40	29.33	27.67
**max-min**	2.14	2.23	2.77	2.82	3.13	2.97	3.72	1.71	2.91	1.92	1.20
**median**	22.44	14.27	26.02	25.91	24.54	24.27	26.04	28.30	26.52	27.85	27.04

Sample \ Reference gene	*Lsi-U6*	*Lsi-18S*	*Lsi-miR166b*	*Lsi-miR167c*	*Lsi-miR167f*	*Lsi-miR166u*	*Lsi-miR398b*	*LsPP2A*
**N sufficient**								
(Bg)R	22.55	14.68	27.14	25.90	25.31	31.04	23.93	26.90
(Wm/Bg)R	22.15	15.45	26.86	26.79	26.00	31.39	25.37	26.85
**N deficient**								
(Bg)R	22.05	14.13	26.31	26.77	24.91	29.59	25.06	25.95
(Wm/Bg)R	21.78	15.41	26.42	26.78	25.36	30.13	24.49	27.15
**P sufficient**								
(Bg)R	22.51	14.17	26.42	26.31	24.77	29.79	24.94	26.77
(Wm/Bg)R	21.97	13.83	26.70	26.84	24.84	30.11	25.01	26.52
**P deficient**								
(Bg)R	22.43	14.77	26.63	26.91	24.48	29.39	24.37	26.67
(Wm/Bg)R	22.94	15.37	27.41	27.85	26.15	31.01	26.38	26.80
**max-min**	1.16	1.62	1.10	1.96	1.67	2.00	2.45	1.20
**median**	22.29	14.72	26.66	26.78	25.11	30.12	24.97	26.78

Sample \ Reference gene	*U6*	*18S*	*miR166b*	*miR167c*	*miR167f*
(Wm/Wm)L	23.64	13.85	26.55	26.63	22.86
(Wm/Sq)L	21.29	13.80	24.56	25.92	21.98
(Wm/Bg)L	21.87	14.69	25.56	25.59	21.88
(Sq)R	24.02	13.71	28.89	29.89	28.18
(Wm/Sq)R	25.03	13.96	28.37	30.37	28.36
(Bg)R	23.38	14.36	29.71	31.32	28.84
(Wm/Bg)R	23.13	13.60	28.65	29.93	27.44
max-min	3.75	1.09	5.15	5.73	6.96
median	23.38	13.85	28.37	29.89	27.44

Wm/Wm: self-grafted watermelon; Wm/Sq: squash-grafted watermelon; Wm/Bg: bottle gourd-grafted watermelon, Sq: non-grafted squash; Bg: non-grafted bottle gourd. "L" represents leaf, "R' represents root.
