# Supplementary material for: Identification of Appropriate Reference Genes for Normalization of miRNA Expression in Grafted Watermelon Plants under Different Nutrient Stresses
Source: PLoS One. 2016 Oct 17;11(10):e0164725. doi: 10.1371/journal.pone.0164725 (PMC5066974; doi:10.1371/journal.pone.0164725)
Supplement: S7 Table — (PDF) [file pone.0164725.s008.pdf]

S7 Table. Expression of *miR85* in watermelon, squash and bottle gourd under nutrient stresses.

Sample \ Reference gene	*miR166b* [a]	*miR167c* [b]	*miR167f* [b]	*miR167c&miR167f&18S&YLS8* [c]
Wm/Wm+N	0.80±0.14	0.91±0.05	0.79±0.13	0.93±0.04
Wm/Wm-N	0.67±0.11	0.50±0.10	0.26±0.04	0.30±0.05
Wm/Wm+P	1.21±0.10	1.30±0.13	1.24±0.11	1.14±0.09
Wm/Wm-P	0.51±0.05	0.73±0.09	0.66±0.07	0.58±0.06
**Sample \ Reference gene**	***miR3511-3p* [a]**	***miR167c* [b]**	***miR167f* [b]**	***miR167c&miR167f* [c]**
Sq+N	0.93±0.12	0.90±0.08	0.96±0.1	0.93±0.08
Sq-N	1.06±0.01	1.94±0.12	2.18±0.07	2.06±0.08
Sq+P	0.79±0.12	0.85±0.08	0.74±0.13	0.79±0.11
Sq-P	0.58±0.17	0.18±0.005	0.19±0.006	0.18±0.003
**Sample \ Reference gene**	***miR398b* [a]**	***miR166b* [b]**	***U6* [b]**	***miR166b&U6* [c]**
Bg+N	1.03±0.04	0.94±0.10	1.03±0.02	0.98±0.05
Bg-N	1.83±0.20	1.29±0.27	0.83±0.10	1.00±0.16
Bg+P	0.90±0.08	0.95±0.12	0.86±0.10	0.90±0.11
Bg-P	1.55±0.12	3.74±0.28	1.63±0.08	2.37±0.12

a, the least stable reference gene; b, single best reference gene; c, the best pair of reference genes.
