# Supplementary material for: Identification of Appropriate Reference Genes for Normalization of miRNA Expression in Grafted Watermelon Plants under Different Nutrient Stresses
Source: PLoS One. 2016 Oct 17;11(10):e0164725. doi: 10.1371/journal.pone.0164725 (PMC5066974; doi:10.1371/journal.pone.0164725)
Supplement: S8 Table — (PDF) [file pone.0164725.s009.pdf]

S8 Table . Expression profiles of target miRNAs retrieved from small RNA-seq.

Name[a]	ID	Length (nt)	Sequence	Normalized reads (Wm/Wm)L	Normalized reads (Wm/Bg)L	Normalized reads (Wm/Sq)L	fold-change (Wm/Bg)L/(Wm/Wm)L	fold-change (Wm/Sq)L/(Wm/Wm)L
Cla-miR164a	conservative_Chr6_1471618	18	TGGAGAAGCAGGGCACGT	35	11	5	0.31	0.14

Name[b]	ID	Length (nt)	Sequence	Normalized reads (Sq)R	Normalized reads (Wm/Sq)R	fold-change (Wm/Sq)R/(Sq)R
Cmo-miR397a	conservative_Chr3_631288	21	TCATTGAGTGCAGCGTTGATG	15	10	0.67

Name[c]	ID	Length (nt)	Sequence	Normalized reads (Bg)R	Normalized reads (Wm/Bg)R	fold change (Wm/Bg)R/(Bg)R
Lsi-miR5148a	conservative_Chr9_2357062	24	GGAGGGGTGCTTGCCTAAGGTCTG	59	23	0.39

Name[d]	ID	Length (nt)	Sequence	Normalized reads (Wm/Wm)L	Normalized reads (Sq)R	Normalized reads (Wm/Sq)R	Normalized reads (Bg)R	Normalized reads (Wm/Bg)R
miR85	unconservative_Chr7_1728984	19	AGGACTTTGAAAAGAAAGA	4	5	5	9	5

a, *Citrullus lanatus* miRNA b, Cucurbita moschata miRNA c, Lagenaria siceraria miRNA

Wm/Wm: self-grafted watermelon; Wm/Sq: squash-grafted watermelon; Wm/Bg: bottle gourd-grafted watermelon, Sq: non-grafted squash; Bg: non-grafted bottle gourd.

"L" represents leaf, "R' represents root.
