# Supplementary material for: Identification of Appropriate Reference Genes for Normalization of miRNA Expression in Grafted Watermelon Plants under Different Nutrient Stresses
Source: PLoS One. 2016 Oct 17;11(10):e0164725. doi: 10.1371/journal.pone.0164725 (PMC5066974; doi:10.1371/journal.pone.0164725)
Supplement: S9 Table — (PDF) [file pone.0164725.s010.pdf]

S9 Table. Relative expression of *Cla-miR164a*, *Cmo-miR397a*, *Lsi-miR5148a*.

Sample	Reference gene	*U6*	*18S*	*miR166b*	*miR167c*	*miR167f*
Cla-miR164a	Wm/Wm	0.97±0.04	1.05±0.05	1.05±0.06	1.09±0.04	1.00±0.04
	Wm/Sq	0.37±00014	1.36±0.1	0.37±0.0038	0.86±0.02	0.66±0.01
	Wm/Bg	0.35±0.02	1.35±0.05	0.58±0.0077	0.71±0.0038	0.62±0.005
**Sample**	**Reference gene**	***U6***	***18S***	***miR166b***	***miR167c***	***miR167f***
Cmo-miR397a	Sq	0.88±0.05	0.84±0.07	0.99±0.02	0.92±0.05	0.86±0.07
	Wm/Sq	0.76±0.05	0.70±0.05	0.33±0.02	0.59±0.02	0.31±0.02
**Sample**	**Reference gene**	***U6***	***18S***	***miR166b***	***miR167c***	***miR167f***
Lsi-miR5148a	Bg	1.11±0.19	0.93±0.03	1.02±0.01	1.07±0.05	0.96±0.02
	Wm/Bg	2.08±0.09	1.24±0.02	2.13±0.13	0.74±0.03	0.96±0.02
